# Can mentorship improve laboratory quality? A case study from influenza diagnostic laboratories in Southeast Europe

**DOI:** 10.1186/s12913-018-3840-0

**Published:** 2019-01-18

**Authors:** Lauren Polansky, Stephanie Chester, Melissa Warren, Tricia Aden, Pamela Kennedy, Stacey Spivey-Blackford, Ann Moen

**Affiliations:** 1 Centers for Disease Control and Prevention, 1600 Clifton Rd, Atlanta, GA 30333 USA; 2 Association of Public Health Laboratories, 8515 Georgia Ave #700, Silver Spring, MD 20910 USA

## Abstract

**Background:**

Strengthening the quality of laboratory diagnostics is a key part of building global health capacity. In 2015, the Centers for Disease Control and Prevention (CDC), the Southeast European Center for Surveillance and Control of Infectious Diseases (SECID), WHO European Regional Office (WHO EURO) and American Public Health Laboratories (APHL) collaborated to address laboratory quality training needs in Southeast Europe. Together, they developed a quality assurance (QA) mentorship program for six national laboratories (Laboratories A-E) in five countries utilizing APHL international consultants. The primary goal of the mentorship program was to help laboratories become recognized by WHO as National Influenza Centers (NICs). The program aimed to do this by strengthening influenza laboratory capacity by implementing quality management systems (QMS) action steps. After 1 year, we evaluated participants’ progress by the proportion of QMS action steps they had successfully implemented, as well as the value of mentorship as perceived by laboratory mentees, mentors, and primary program stakeholders from SECID and WHO EURO.

**Methods:**

To understand perceived value we used the qualitative method of semi-structured interviews, applying grounded theory to the thematic analysis.

**Results:**

Mentees showed clear progress, having completed 32 to 68% [median: 62%] of planned QMS action steps in their laboratories. In regards to the perceived value of the program, we found strong evidence that laboratory mentorship enhances laboratory quality improvement by promoting accountability to QMS implementation, raising awareness of the importance of QMS, and fostering collaborative problem solving.

**Conclusion:**

In conclusion, we found that significant accomplishments can be achieved when QA programs provide dedicated technical mentorship for QMS implementation. Since the start of the mentoring, Laboratory “B” has achieved NIC recognition by WHO, while two other labs made substantial progress and are scheduled for recognition in 2018. In the future, we recommend that mentorship is more inclusive of laboratory directors, and that programs evaluate the amount of staff time needed for mentorship activities, including lab-based assessments and mentoring.

**Electronic supplementary material:**

The online version of this article (10.1186/s12913-018-3840-0) contains supplementary material, which is available to authorized users.

## Background

Within the World Health Organization’s Global Influenza Surveillance and Response System (GISRS), National Influenza Centers (NICs) are responsible for collecting, evaluating, and sending viral specimens to WHO Collaborating Centers (WHO CCs). Annually, the WHO CCs identify the most representative influenza strains for use in global vaccine production. NICs also share the types of influenza viruses circulating weekly via the virologic monitoring platform, WHO FluNet. To become a NIC, an institution must be designated by the country’s ministry of health and formally recognized by WHO through an assessment process [1, 2]. As one of the six WHO CCs for Influenza, the Centers for Disease Control and Prevention (CDC) aims to strengthen global influenza surveillance by improving laboratory quality and increasing the number of laboratories that contribute to GISRS as NICs [3].

**Fig. 1:**
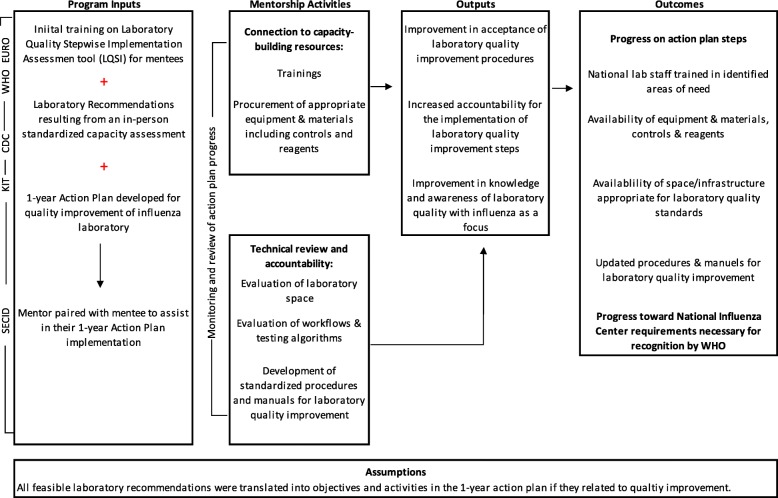
Description of Mentorship Program

To further these efforts, in 2005 CDC collaborated with the Association of Public Health Laboratories (APHL), and together, they established a group of U.S. public health laboratory experts to serve as international consultants with specific expertise in laboratory systems and influenza laboratory diagnostics [4]. In 2015, seeking to address quality improvement needs in Southeast Europe, the CDC, the Southeast European Center for Surveillance and Control of Infectious Diseases (SECID), WHO European Regional Office (EURO) and APHL formed a collaborative mentorship program. Together, they developed a quality management (QMS) mentorship framework that utilized APHL consultants as mentors. The primary goal of the mentorship program was to help national influenza laboratories become National Influenza Centers (NICs) officially recognized by WHO. By mentoring laboratories through the process of implementing QMS action steps it was hoped that laboratories could close quality gaps, and eventually operate as a NIC within the GISRS network (Fig. 1).

Six laboratories (designated A-E) from five countries were included in the program: Albania, Bosnia and Herzegovina (Sarajevo & Banja Luka), Kosovo, Macedonia and Montenegro. One country, Albania, was already a WHO recognized NIC; however, due to challenges with maintaining capacity and quality improvement, implementing partners in the region agreed that the lab could also benefit from mentorship.

Prior to the mentorship initiation the laboratories participated in a training conducted by KIT (Royal Tropical Institute) and EURO on the WHO Laboratory Quality Stepwise Implementation (LQSI) tool which guides laboratories toward implementing a QMS in accordance with the International Organization for Standards (ISO) 15,189 requirements [5, 6]. A mentor and mentee can download specific LQSI step-wise implementation checklists (and associated standardized templates and examples) based on the LQSI phases and modules that are of priority [6]. In addition, the mentee’s laboratory was also assessed by their mentor using the CDC-APHL *International Influenza Laboratory Capacity Review Tool* that provided them lab-specific recommendations (hereon referred to as “assessment recommendations”) at the launch of the mentorship project [7]. The capacity review tool was designed with the capacities for meeting NIC terms of reference in mind. Assessment recommendations that result from the tool help to identify potential areas for technical assistance specifically related to influenza diagnostics. By translating both the LQSI tool and assessment recommendations into QA mentorship action plans (heron referred to as “action plans”), the laboratories can work simultaneously on QMS improvements and capacity building that contributes to meeting or maintaining NIC terms of reference (Fig. 1, Table 1). The plans vary based on each laboratory’s unique context. They serve as a framework for the program to monitor and evaluate progress, and whether they may be ready to perform adequately as a NIC in the region. A comparison of focus areas for the NIC Terms of Reference (ToR), CDC-APHL International Influenza Laboratory Capacity Review Tool, and WHO Laboratory Quality Stepwise Implementation (LQSI) tool are provided in Table 1 [2, 6, 7].

**Table 1:** Comparison of focus areas in the NIC terms of reference (ToR), CDC-APHL International Influenza Laboratory Capacity Review Tool, and the WHO Laboratory Quality Stepwise Implementation (LQSI) tool

General Conditions for NICs: Terms of Reference	CDC-APHL International Influenza Laboratory Capacity Review Tool: Nine modular section	WHO Laboratory Quality Stepwise Implementation (LQSI) tool: Four modular phases:
Serve as a reference laboratory for influenza in their country Serve as a technical resource on influenza-related matters for their national authority Serve as a key point of contact to WHO on issues related to influenza in their country Adhere to their national and/or international biosafety standards for work with influenza viruses Adhere to national and international regulations on the transport of dangerous goods (Class/division 6.2)4 when shipping clinical specimens and/or virus isolates Meet quality requirements of national or international quality standards, as applicable, and participate in external quality assessment programmes (EQAP) provided by WHO to GISRS Maintain a high level of technical proficiency by participating in training provided by GISRS	Laboratory Contact Information General Laboratory Specimen Handling, Collection, and Reporting Virology Laboratory Molecular Biology Laboratory Laboratory Safety and Biosafety Quality Assurance / Quality Control Equipment Training	Ensuring that the primary process of the laboratory operates correctly and safely Controlling and assuring quality and creating traceability Ensuring proper management, leadership and organization Create continuous improvement and prepare for accreditation

### Mentor selection

CDC and APHL selected four mentors to mentor six labs, resulting in two mentors doubling up on mentees. Mentors were able to commit to 12 months of engagement, and had a minimum of 15 years’ experience in public health laboratories. Additionally, all mentors had conducted international influenza laboratory capacity reviews, and therefore had a solid grounding in international capacity building engagement, expertise, culture and leadership.

### Mentee selection

All participating laboratories were formerly designated by their respective ministries of health to perform influenza virological surveillance. Accordingly, each ministry of health nominated influenza laboratory staff members to participate in this program. The size of each laboratory varied from just two staff to 15 staff, with most staff rotating to other areas of the laboratory as needed. Likewise, the total number of specimens processed for influenza results during the evaluation period varied from 82 specimens to 775, with two laboratories having no results available on the GISRS open-access, global virologic monitoring platform, FluNet (Table 2). All of the laboratories selected for this program also participate in the surveillance and control of communicable disease activities of the Southeastern Europe Health Network, coordinated by the Southeast European Center for Surveillance and Control of Infectious Diseases (SECID). Additionally, SECID together with all laboratories contribute to the implementation of the project “Surveillance and Response to Avian and Pandemic Influenza by National Health Authorities of Countries of South East Europe” supported through a cooperative agreement with the United States Centers for Disease Control and Prevention that also provides technical support coordinated with the WHO/Europe.

**Table 2:** Laboratory capacity

Country	Percent of specimens reported positive for influenza to WHO FluNet during evaluation period (July 2015 - June 2016)	Number of laboratory staff performing RT-PCR for influenza
A	33% (*n* = 214/ 674)	5 staff trained in PCR. Staff members are cross-trained and rotated to different testing areas of the laboratory
B	40% (*n* = 176/441)	10–12 full-time staff perform PCR in the influenza laboratory. Staff members are cross-trained and rotated to different testing areas of the laboratory
C	31% (*n* = 242/775)	3 staff perform part-time PCR in the influenza laboratory 2–3 h a day during influenza season from October to April.
D	No report to WHO FluNet	2 staff routinely perform PCR testing.
E	No report to WHO FluNet	2 staff routinely perform PCR testing.
F	28% (*n* = 23/82)	7 full-time staff perform PCR in the influenza laboratory. Staff members are cross-trained and rotated to different testing areas of the laboratory

### Mentorship

Mentors discussed challenges and progress with mentees on a monthly basis via email and telephone, and tracked progress using the action step Gantt chart that they co-developed together following the LQSI training. To facilitate communication and sharing of documents, EURO set up an EZCollab document-sharing website for mentors and mentees.

We conducted an internal, formative evaluation of the program (July 2015—June 2016) to answer the following evaluation questions:

- To what extent were QA action plans achieved?
- What was the perceived value of a mentorship program in the Southeast European region?

This paper responds to these questions through a discussion of the progress and observations made after the first year of mentorship.

## Methods

To evaluate progress, we measured the percentage of the action plan steps that were in a status of completed, in progress, and without progress as reported and validated by mentors and mentees after one year. We utilized qualitative methods as a formative approach to evaluating the perceived value of mentorship and the lessons learned [8–10]. To support the heterogeneity of the differing laboratory contexts, we utilized grounded theory, which allows for the unique experiences of each laboratory and mentor to be discussed and facilitated through a standard methodology [11]. Over 5 days, a trained qualitative evaluator from CDC who was not familiar with the program, and did not participate in the program, conducted face-to-face, semi-structured interviews with the six laboratory mentees and four mentors (26 questions), with a stakeholder from WHO regional office for Europe most directly involved in the program (14 questions) and with one key stakeholder from SECID who is responsible for coordinating capacity building for influenza infectious disease surveillance in the region (14 questions). This resulted in a total of 282 questions coded [6 mentee transcripts with 26 questions, 4 mentor transcripts with 26 questions, and 2 stakeholder transcripts with 14 questions] (Additional file 1: Appendix A. Example Survey Questions). APHL and CDC vetted the interview questions among laboratory subject matter experts. The general topic guide included the following: 1) development of the quality improvement plan, 2) connection to and follow-up with APHL mentor/mentee over the course of the year, 3) implementation guidance and support, 4) long-term results of the project & progress towards NIC requirements and/or designation, 5) lessons learned about technical mentorship for laboratory quality improvement. A note-taker who also did not participate in the program, recorded written transcripts of the interviews. Interview times ranged from 60 to 86 min. This was a convenience sample specifically chosen for program evaluation; therefore, all program participants were interviewed and there was no saturation point [12, 13].

A program evaluator with qualitative analysis training independently coded transcripts using grounded theory to identify themes, and to later analyze and interpret them through content analysis [11, 12, 14] . Cleaned transcripts were organized into Microsoft Excel by respondent and question number, and codes and sub-codes were manually entered for each question. Due to the hand-written nature of the notes, we uncovered instances where some of the interview notes did not provide enough of the discussion to accurately code. In these cases, we coded the response as ‘missing’ rather than attempting to guess. To validate the reliability of coding, a second-coder was trained to independently code the transcripts in a separate Excel workbook. The question codes across the 12 transcripts (*N* = 6 mentees, 4 mentors, and 2 stakeholders) were compared, and resulted in an agreement of 86% across all interview transcript questions (*n* = 241/282). The 14% (*n* = 41/296) discrepant codes were resolved through discussion. We opted for using % comparison as a measure of reliability, with two coders directly coding all questions independently, because our purpose was coming to consensus in interpretation for all themes in our evaluation, rather than determining the accuracy of applying finalized, pre-set codes to a larger data set [12–14].

### Evaluation assumptions

It is important to note that the number and type of action steps developed for each laboratory differs. This necessarily reflects differing phases of development, capacity, and resources. Rather than emphasizing the same action steps across laboratories, all mentors emphasized choosing steps that were a priority and were feasible to achieve in one year, based on the laboratories unique situation (Fig. 1).

## Results

### Prioritizing which areas to improve

The total number of action steps from each national lab ranged from 9 to 62 [median = 22]. A median of 55% (IQR 43–67%) of the assessment recommendations were translated into action steps (Table 3).

**Table 3:** Status of action steps after one year

Laboratory	Number of action steps	% of assessment recommendations that were incorporated as an action step	Status of action steps after one year		
	N	% (n/N)	Complete % (n/N)	In Progress % (n/N)	No Action % (n/N)
A	9	78% (7/9)	44% (4/9)	56% (5/9)	0% (0/9)
B	22	50% (11/22)	64% (14/22)	27% (6/22)	9% (2/22)
C	22	27% (6/22)	64% (14/22)	23% (5/22)	14% (3/22)
D	22	64% (14/22)	68% (15/22)	14% (3/22)	18% (4/22)
E	22	86% (19/22)	59% (13/22)	36% (8/22)	5% (1/22)
F	62	21% (13/62)	32% (20/62)	40% (25/62)	27% (17/62)

When asked openly about what helped mentees and mentors to choose action steps for quality improvement, the LQSI process was stressed by the majority (*n* = 5/6 mentees; and 2/4 mentors). We heard that “*The LQSI tool (Laboratory Quality Management tool) helped to guide the process (it was the Bible)”* and that it was *“useful for their staff members to write the action plan about how to train other laboratorians to help in the influenza laboratory. [We brought] back a retired person to assist in the influenza laboratory - the action plan was the driver of that change.” (Mentee).* Another mentee explained that, *“The APHL-CDC International Influenza Laboratory Capacity Review is more science based, so using the LQSA tool helped ‘pinpoint’ issues with process.”* When probed, all laboratories, regardless of NIC status or capacity, reported that the LQSI tool was advantageous and no mentor or mentee reported it disadvantageous. Equally stressed as important for choosing action steps were the assessment recommendations (*n* = 4/6 mentees; 3/4 mentors). When probed, one of the mentors reflected that, *“The capacity lab assessment helped to frame the ideas to help push along. If the mentorship program were recreated, a laboratory assessment is critical or at least review of a previous assessment.”* Upon further questioning, mentees reflected that the assessment recommendations helped influence the “credibility” of the action plan itself given the more extensive understanding of the national laboratory by the mentor and mentee (*n* = 5/6 mentees).

### Fostering accountability was key to progress made

After one year, 32 to 68% of QMS action steps were completed [median: 62%] (Table 3). Due to the translation of 21 to 86% of assessment recommendations into action steps, a median of 58% of assessment recommendations were also completed through the process of completing action steps.

All mentees (n = 5/6, 1 missing) and mentors (*n* = 4/4) felt that mentorship was critical to fostering implementation of QMS through accountability. *“Without mentorship the accomplishments would not have happened. It definitely made [us] more accountable, [and] pushed [us] to complete tasks” (mentee).* Each mentor further stressed the value of accountability. “*The mentorship program was a good accountability measure because they know every month they will be asked about progress,*” and “[*the] advantage of the mentorship program [was that it] laid out expectations and a timeline. These two things are very important” (mentor).* Mentees highlighted the value of having regular discussions with their mentors (*n* = 4/6 mentees), and designating a quality assurance manager or team inside of the laboratory that was responsible for advancing actions for quality improvement (*n* = 2/6 mentees). In regards to the differences between being a mentor and a laboratory assessor, we heard that “*as an assessor you come in and make recommendations and [the labs] don’t have anyone to go to get the recommendations accomplished, versus mentorship, which gives them recommendations and someone to work with to accomplish the recommendations.”* Another mentor saw the value of mentorship versus laboratory assessment as helping to shape the acceptance of quality management systems*, “it help[s] the country understand why this all is important and not just a recommendation” (mentor).*

The action items were organized in a Gantt chart for monthly progress reporting, which according to all mentors was effective for helping them to visually track progress and anticipate future needs from their mentees (*n* = 4/4 mentors). WHO and SECID stakeholders were satisfied with the content of these reports and felt that it enabled effective follow-up (*n* = 2/2). All mentees who responded felt that the communication with their mentor, whether weekly, bi-weekly, or monthly was satisfactory, while two of the four mentors expressed a desire to have communicated more often. Mentors struggled to maintain monthly follow-up on progress, particularly when laboratory staff were on vacation. They described all communication as “mentor-initiated,” and expressed a desire for laboratories  to take more ownership of the process. Three of the mentees agreed that follow-up was mentor initiated, while two felt equal initiation (*n* = one missing from transcript).

### The program helped address implementation challenges

There were two common themes regarding challenges to implementing the action plans. The first was the need for cultural and behavioral changes within the laboratory. Sub-themes described were a lack of wider staff involvement and motivation to perform routine quality management. One explanation given was that it is not yet being institutionalized as a part of the laboratorians’ jobs (*n* = 3/6 mentees, *n* = 1/4 mentors). In close connection, the lack of support from management and decision makers to implement quality improvements was another important sub-theme (*n* = 3/6 mentees, *n* = 1/4 mentors). One mentee commented that, “*getting people involved and to understand, especially at higher levels, is a challenge. Overcoming is hard and [I] continue to try. The mentoring program was a kick off point to build the culture and it would be helpful to get upper management involved from the beginning.”* The second theme regarding challenges was management of resources (*n* = 4/6 mentees, 1/4 mentors) including shortages of staff and routine training for staff, reagent procurement, and equipment maintenance.

Most mentees (*n* = 4/6 mentees, *n* = 2 missing) and mentors (*n* = 3/4 mentors, *n* = 1 missing) reported that the mentor program helped to address these challenges. Specific sub-themes mentioned were advocacy and awareness raising (*n* = 2/6 mentees, *n* = 1/4 mentors), improvement of standard operating procedures (*n* = 1/4 mentor), providing general guidance and accountability on how to approach quality improvement implementation (*n* = 2/6 mentees), and getting internal buy-in for QMS support through SECID’s involvement (*n* = 1/6 mentees, *n* = 1/4 mentors). Mentorship itself, in terms of being connected to a mentor, stood out as the most common “tool” mentioned for QMS problem solving (*n* = 6/6 mentees). Sub-themes were mentors’ abilities to draft a letter to the director to raise awareness of issues (*n* = 1/6), perform laboratory assessments (*n* = 2/6), solve technical problems (*n* = 2/6) and the mentee’s accountability to their mentor for deadlines and actions related to quality improvement plans (*n* = 2/6). Both mentees and mentors described SECID’s role as very influential due to their pre-existing relationships with the ministries of health and laboratory directors. Specific sub-themes related to their value as collaborators were creating an open, extended network of partners, logistic support of laboratory purchases connected to NIC functions, and support of internal management within the laboratory (*n* = 4/6 mentees; *n* = 2/4 mentors). *“SECID was really important. [Laboratory staff] are used to working with SECID and already had the relationship” (mentor).*

Before the program began, mentees reported that it was “somewhat difficult” to address quality improvement. After one year, all mentees felt it had become somewhat easy to very easy (*n* = 6/6 mentees). This was validated by mentors also felt each laboratory moved from an area of “somewhat difficult” to “somewhat easy” (*n* = 4/4 mentors). Table 4 presents the types of progress described by mentees or mentors. *“[It’s the] tip of iceberg – no one spoke of laboratory acquired infection prior to this program and also biosafety. Culture and behavior change [occurred] overall”* (mentee). In addition, mentees found the program to be very impactful (*n* = 3/6) or critical (n = 3/6) to their quality improvement. One mentor verified this stating that, *“Laboratories are seeing progress and are able to accomplish items,*” and *“[there is] overall improvement in processes. [They] now have Standard Operating Procedures in place [which is] a major improvement [in] the actual flu surveillance rather than just diagnostic [methods]”* (mentor). Mentees also reported improved skills through the mentorship program. Sub-themes included skills in management, organization and evaluation, and an increased ability to identify and plan for ways to address quality improvement issues. “S*uccess has been better having CDC, APHL, and SECID behind [me] for credibility; [I] am more confident, and understanding how other labs [do quality improvement] is helpful” (mentee).*

**Table 4:** The types of progress observed by mentees or mentors

Mentee Observations	n/N	Lab (A-E)	Mentor Observations	n/N	Lab (A-E)
Creation of appropriate laboratory space & acquisition of appropriate equipment	2/6	D, F	Improved quality management system	2/4	D, E, F
Development of quality management knowledge/skills and building a network	1/6	D	Understanding the importance of quality management	1 /4	F
Increased awareness of quality management practices	1/6	C	Improved laboratory testing and surveillance processes	1/4	C
Improved lab capacity and confidence in safety of the lab	1/6	E	Improved confidence in the ability to accomplish action items	1/4	D, E
Starting influenza virus isolation	1/6	B	Progress toward National Influenza Center recognition	1/4	A, B
Development of Standard Operating Procedures (SOPs) & Quality Assurance (QA) Plan	4/6	A, B, F, C

**Table 5:** Sections of SLIPTA

Documents and Records	
Management Reviews	
Organization and Personnel	
Client Management and Customer Service	
Equipment	
Internal Audit	
Purchasing and Inventory	
Process Control and Internal and External Quality Assessment	
Information Management	
Corrective Action	
Occurrence Management and Process Improvement	
Facilities and Safety	

### Achieving National Influenza Center (NIC) designation

When we collected data for this evaluation, virus isolation capabilities, such as cell culture, were a required NIC function. In relation, mentees stated that the area of most concern for achieving and maintaining NIC requirements was virus isolation [2]. All of the action plans included at least two action steps aimed at improving virus isolation. The overall progress made across the different NIC requirements was reported as difficult to discern by both mentees and mentors (*n* = 6/6 mentees, 4/4 mentors). Never the less, mentors felt that the program was somewhat important to the laboratory’s ability to make overall progress toward NIC designation. At the time of data analysis, in addition to NIC-ALB which annually maintains the status of WHO recognition, Laboratory “B” achieved WHO NIC recognition. Three other laboratories have also made substantial progress with Laboratory “F” scheduled for recognition in 2018 and Laboratory “D” and “E” in the first quarter of 2019. After the evaluation period, WHO decided to eliminate virus isolation from being a required part of the NIC terms of reference going forward, which could impact the way in which action steps related to virus isolation are approached and prioritized in the future [15].

### Improvements beyond influenza diagnostics

The impact of quality improvements extended beyond influenza diagnostics. Four mentees mentioned changes in other laboratories (*n* = 4/6) and this was validated by each mentor (n = 4/4). *“[Quality improvements] expanded to the entire virology department and helped to identify issues in measles. The QA was designed to address the whole laboratory from the beginning”* (mentee). *“[We] have just started training the whole department on QMS and training on biosafety and biosecurity is [the] next step. Most [staff] do not have this knowledge because it is not in [the] school curriculum. [So] they are now instituting an internal training process in the department, and QMS is a part of the material and curriculum” (*mentee).

### Recommendations from laboratories, mentors and WHO

To improve the QMS laboratory mentorship programs, mentees suggested organizational changes that support laboratory quality improvement such as management support of laboratory staff conducting QMS activities. This included supporting team building for quality assurance and clearer roles and responsibilities for staff. Mentors recommended scheduling a minimum time commitment from the laboratory devoted to quality management activities, such as 20% of staff time per week. Mentors also recommended that the program begin with a laboratory assessment that is re-assessed after 1–2 years, as well as equipping the document sharing platforms, such as EZCollab, with all reference documents at the beginning of the program. Stakeholders from WHO and SECID commented that the multi-partner approach worked well. They defined this approach as working with a WHO regional office through the existing WHO GISRS network, having laboratory mentorship from trained APHL consultants, focusing on national public health laboratories with an interest in improving capacity, having a technical implementation partner such as CDC, and having a local, grant-funded, regional collaborator with pre-established relationships with decision makers such as SECID. In this case, *“It [is] a combination of mentoring, the CDC Cooperative Agreement funding with SECID through which countries get resources to buy reagents, and the support from WHO in terms of training. It is really a constellation of partners and support. Because if you’re assigned a mentor without the other two partners, you have a recommendation, but not the means to do it” (stakeholder).*

## Discussion

We describe our evaluation in relation to two questions: (1) to what extent were QA action plans achieved?, and (2) what was the perceived value of a laboratory quality mentorship program?

In evaluating our first question, we found that each laboratory created action plan steps (median 22 per laboratory) geared to improve laboratory capacity and gain NIC status, and 61% of planned action steps were complete after one year, demonstrating clear progress. In regards to our second question, the program enhanced accountability for implementing planned actions and increased awareness of the importance of quality management processes in a laboratory, extending beyond the influenza laboratory. We discovered that mentees and mentors perceived the mentorship program as beneficial to addressing barriers to laboratory quality improvement. Moreover, laboratory "B" achieved the goal of NIC recognition, while laboratory "F" earned an official assessment in 2018, followed by planned assessments for laboratories "D" and "E" in 2019.

Laboratory quality improvement seems to be a growing area within international laboratory capacity building. In a 2010 review of published reports, major donor interviews, and case studies of laboratory systems in three resource-limited countries Olmsted et al. recommends that, “host countries and their external partners should incorporate laboratory standards, comprehensive quality systems, and even goals for accreditation in their plans for laboratory development” [16]. Although NIC recognition is not comparable to accreditation, it does provide a standard for operation, and creates a network and connection to other laboratories and external agencies through WHO GISRS.

Three other international QMS mentorship models also offer lessons learned [5, 17, 18] . Peronne et al. implemented a QMS mentorship model in Southeast Asia to strengthen the quality and capacity of Cambodian hospital laboratories [5]. By recruiting and training four local laboratory technicians, they mentored staff from 12 hospitals on QMS practices, and used the LQSI tool as their primary assessment tool translated into Khmer – one of the local languages. According to Peronne et al. “project staff reviewed the laboratories’ progress and challenges in weekly conference calls and bi-monthly meetings with focal points of the health ministry, participating laboratories and local partners” [5]. After 18 months, 12 laboratories had completed 74–90% of the 104 activities in phase 1, 53–78% of the 178 activities in phase 2, and 18–26% of the 129 activities in phase 3. They highlight the importance of imbedding mentorship into the worksite and using local languages; however, caution that for the majority of laboratories in developing countries, accreditation, emphasized in the final phase of the LQSI tool, is not feasible due to resource and capacity constraints, potentially limiting the scope and effectiveness of assessment tools like the LQSI alone.

In the African region, for example, Alemenjii et al. points out that accreditation is still not cost-effective for the majority of laboratories [19]. In a review of laboratory strengthening in sub-Saharan Africa, they found that achieving practical and sustainable laboratory accreditation was a major challenge owing to “lack of leadership, attention, resources and commitment” [19]. Of the over 340 accredited laboratories in Africa, they find that 312 (92%) were located in South Africa alone which is a country with a national accreditation body; the South African National Accreditation System [20]. While utilizing local languages and regional staff should absolutely be considered in easing the cultural and technical language barriers of global laboratory mentorship, our program’s hybrid approach of combining the LQSI approach with a standard assessment of the influenza laboratories without the emphasis on accreditation could offer an advantage.

Another program, which can be compared, is the Stepwise Laboratory Quality Improvement Process Toward Accreditation (SLIPTA). Maruta et al., describes a SLIPTA mentorship program at three district and one central laboratory in Lesotho, which aimed to help with accreditation, a similar focus to Peronne et al. [5, 17, 18]. Their model had a mentor from the Southern Africa region fully embedded within the operations of each of the laboratories for an initial six-week and then a follow-up four-week visit, separated by 6–8 weeks. Quality improvements measured at baseline using the SLIPTA check-list improved significantly in all four laboratories after 10 weeks, with the central laboratory progressing significantly after just 5 weeks (from 44 to 57% completed steps; *p* = 0.021) [18]. Like our model, the SLIPTA model formulated action plans jointly with mentors using a standardized tool, in their case the SLIPTA checklist that resulted in a type of Gantt chart with activities, responsible person, timeline, and review dates, which became a working document with a defined improvement path. The major difference in the SLIPTA program compared to ours was the presence of the mentor in the lab for 10 weeks following the baseline assessment, versus a one-week baseline assessment visit with associated recommendations. The SLIPTA mentor also mentored the entire lab, not just one mentee, and offered group discussions and presentations for the entire laboratory once every week on topics identified by the baseline assessment. They also held staff meetings to give advice and coaching on issues arising from the lab.

While this immersed, work-based approach could be an advantage of the SLIPTA mentoring program; one of the major reported drawbacks is cited by Yao et al. who cautions that areas not addressed in depth by SLIPTA (Table 5) include quality control principles and practices, writing standard operating procedures, biologic safety, and quality assurance manager training. However, improved standard operating procedures and biosafety actions have been identified as key barriers for the improvement of international laboratory capacity through the CDC-APHL capacity review tool [17, 21]. The sustainability of changes facilitated during intensive mentorship periods with a mentor present is also something to consider.

These studies combined with our formative evaluation results, yield four elements to highlight when building laboratory QMS and capacity through mentorship. First, it is critical to have a standardized tool, like the LQSI, to guide laboratorians through identifying possible quality management actions with standardized templates and examples for those actions. All mentees, regardless of their laboratories’ NIC status or capacity, reported that the LQSI tool was advantageous in helping them develop their action plans and did not report any challenge or disadvantage to using it.

Second, providing a standardized and thorough baseline assessment of the laboratory’s general capacity through a tool like the *CDC-APHL international influenza laboratory capacity review tool* is a necessary part of action plan development. As discussed, an advantage to combining these two tools and approaches is that the focus can remain on building technical levels of laboratory quality, such as SOPS and biosafety, rather than on accreditation, which may not be feasible in many developing contexts, and is beyond the control of individual laboratories [5, 17–19]. An immersive, work-based opportunity like the baseline assessment supports the third element to highlight - connecting the laboratory to a mentor whom they trust with appropriate time and expertise for follow-up of planned actions. We recommend face-to-face observation and support within a mentee’s laboratory environment for establishing realistic expectations and a trusting line of communication for follow-up. The length of time, frequency, and the type of staff included in a mentor’s laboratory assessment visit should be evaluated [3, 4, 18].

Fourth, to support QMS problem solving, it is *as* important to have the awareness and support of upper management, as it is to link the laboratory to effective implementing partners in the region, as some factors are beyond the control of the mentees, such as laboratory space and procurement issues. Mentors and stakeholders in this evaluation noted that a lack of awareness and support for issues beyond their control might have contributed to action steps falling behind schedule, and this was the most common explanation of heterogeneity in progress made. While some mentees created small organizational changes such as establishing a quality assurance officer or team, the fact that mentorship occurs at the level of mentees, creates some challenges for organizational change. Similarly, Peronne et al. also reports that several management challenges exist around enforcing habits of quality assurance. They highlight the need for strong leadership from laboratory directors and hospital management, whom they explain may have assumed their positions due to their technical skills or seniority, and not had a chance for appropriate laboratory management experience [5]. One recommendation from the SLIPTA evaluation, which may help implementation, is paying more attention at the start of the program to ensure that participating laboratories have available, “a national laboratory strategic plan and policy, a laboratory director with decision making power, a quality assurance manager, and that participants are committed to the same job responsibilities during the program time frame” [18]. It is also important to consider creating an action plan that is flexible in allowing laboratories and mentors to adjust the schedule, so that when issues occur in one area, they can shift focus to another, until addressing the original issue.

## Limitations

While the progress on overall quality improvement was easy for mentors and mentees to discern through the action plan, they reported that understanding their progress towards the specific NIC requirements was more difficult for them to discern (*n* = 6/6 mentees, *n* = 4/4 mentors). Therefore, it is important that in future mentorship programs, clear steps and standards for NIC requirements are understood for better performance monitoring.

## Conclusions

The regional mentorship program for influenza diagnostic laboratories in Southeast Europe serves as an initial proof of concept that significant accomplishments are achieved when quality implementation programs provide mentorship. We found that 61% of planned action steps were complete after 1 year. The mentorship program enhanced accountability for implementing planned actions and increased awareness of the importance of quality management processes in a laboratory, extending beyond the influenza laboratory. Moreover, both mentees and mentors perceived the mentorship program as beneficial to addressing barriers to laboratory quality improvement, and stakeholders were satisfied with the progress made. Since the start of the mentoring program, Laboratory “B” has achieved NIC recognition, while two other labs made substantial progress and are scheduled for recognition in 2018 (Laboratory “F”) and the first quarter of 2019 (Laboratory “D” and “E”). Meanwhile, NIC-Albania annually maintains the status of WHO recognition. In reaction, CDC and APHL have launched a new mentorship program with ten countries in the Africa region. Based on lessons learned from this evaluation, laboratory capacity assessments and LQSI tool training are continuing to help action plan development. To improve upon the program, they introduced a standardized template to support regular reporting of progress and challenges, including specific items for NIC terms of reference, and a collaborative online platform with shared resources. Performance monitoring that relates to NIC designation, such as timely sharing of clinical specimens with WHO Collaborating Centers for seasonal influenza vaccine strain selection and sharing of weekly virologic monitoring data through WHO GISRS, are being actively monitored to give feedback to mentors and mentees along the course of the program. Although challenging, the new program has asked senior leaders within the national laboratories to not only formally acknowledge their staffs’ participation in the program, but to do so by committing to support the time and work it requires of their staff and to review updates reported from the program.

## Additional file

Additional file 1: Summary of Interview Questions. Examples of the interview questions used during the evaluation. (DOCX 28 kb)

## Abbreviations

APHL: Association of Public Health Laboratories
CDC: Centers for Disease Control and Prevention
EURO: WHO Regional Office for Europe
GISRS: Global Influenza Surveillance and Response System
ISO: International Organization for Standards
LQSI: Laboratory Quality Stepwise Implementation Tool
NICS: National Influenza Centers
QA: Quality Assurance
QMS: Laboratory quality management systems
SECID: South East European Center of Infectious Disease
WHO CCs: WHO Collaborating Centers
