# Supplementary material for: Can mentorship improve laboratory quality? A case study from influenza diagnostic laboratories in Southeast Europe
Source: BMC Health Serv Res. 2019 Jan 18;19:49. doi: 10.1186/s12913-018-3840-0 (PMC6339419; doi:10.1186/s12913-018-3840-0)
Supplement: Supplementary file 1 — Summary of Interview Questions. Examples of the interview questions used during the evaluation. (DOCX 28 kb) [file 12913_2018_3840_MOESM1_ESM.docx]

**Appendix A: Questions and prompts used during the interview for mentees, mentors, and stakeholders**

**Questions for Mentees:**

**Development of the quality improvement plan**

**[Interviewer: *So I understand that you have been working on completing action steps over the past year. I want to talk a little bit about the development of the quality improvement plan, to learn what worked well and how we might be able to improve the process in the future*]**

1. **How did your laboratory decide which action steps to focus on over the past year?**

**[Interviewer Transition*: I understand that you participated in the LQSI training.… Probe: Can you tell me a little bit more about that?***

1. **Did you notice any advantages to using the LQSI training & tool to develop your action plan?**

***Probe: Can you tell me a little bit more about that?***

***Probe: So everything was clear to you? And everything seemed relevant?*
What about any disadvantages?**

1. **Were there any other assessments, evaluations, or internal laboratory findings that went into your action planning?**

***Probe: Can you tell me a little bit more about that?***

***[Interviewer Transition: If CDC/APHL assessment NOT mentioned: I understand that your lab was also assessed with the CDC/APHL capacity review tool…can you tell me a little more about that?]***

**3b. Did you do the CDC/APHL International Influenza Laboratory Capacity Assessment before or after making your action plan?...If before, how much did you rely on those recommendations in prioritizing activities over the past year?**

***Probe: Can you tell me a little bit more about that…what parts were helpful? Why you didn’t/did use it?***

1. **If you hadn’t received mentorship through this project, how likely would it have been for you to develop & track a quality improvement plan?**

***Probe: In what ways did the project help you prioritize action steps?***

**Connection to and follow-up with APHL mentor over the course of the year**

***[Interviewer: It would be nice to talk some about how you and your mentor have communicated and followed up over the past year]***

1. **How would you describe the communication & follow-up between you and your mentor over the past year**?

**Too Frequent, Just Right, Not Frequent Enough**

***Probe: Why was that? Who reached out to who first?***

1. **About how often did you and your mentor communicate (on average)?**

**Weekly, Every Few Weeks, Monthly, As needed**

***Probe: who typically initiated communication? For what reason?***

1. **What was the best way(s) to communicate with your mentor over the past year?**

**Email , Skype, Telephone, Other**

***Probe: Why was that?***

***Probe: What types of challenges did you experience?***

1. **How helpful was the monthly check-in with your mentor to review & make progress on action steps for your laboratory?**

***Probe: what would have made the process better for you?***

***Probe: is there anything you’d change to make the process more helpful?***

***Probe: is there anything you’d change to make the process more helpful?***

1. **How clear was the process for developing and sending monthly reports to your mentor?**

***Probe: why?***

**9a. So on a scale from 1 to 4, 1 being very difficult and 4 being very easy, you’d say reporting monthly to your mentor was…**

**1.** very difficult**, 2.** Somewhat difficult**, 3.** fairly easy**, 4.** Very easy

***Probe: why?***

***Probe: Can you think of anything that would make reporting to your mentor easier, clearer, better, or more useful for you?***

1. **Is there anything that you would change about the follow-up processes with your mentor?**

**10a. In regards to year 2, do you think a standard, reporting template improve the utility of information you receive?**

***Probe: And if so, do you have any suggestions?***

**Implementation guidance & support**

***[Interviewer: Now I’d like to talk about implementation of the plan & how you were supported by your mentor]***

1. **Did you use EZCollab as a way to access documents? [let them answer]**

***Probe: If no, was there any reason why not?***

1. **Were there any documents/resources that your mentor shared outside of EZCollab that helped in the development of your own material?**

**Probe: How often did they share documents outside of the EZCollab system?**

***Probe: Was it as you requested them or did they share things proactively?***

1. **Were there any specific documents that helped in the development of your own material?**

***Probe: what were they? Did you access them on EZCollab or were they sent directly?***

1. **What were some of the challenges you faced in implementing your quality improvement plan?**

***Probe: Were the challenges expected?***

***Probe: What helped you to overcome the challenges? ___________***

1. **Can you think of anything that has helped with challenges that you faced over the past year**?

***Probe: Can you give an example or explain?*** __________

1. **What was the most helpful type of support you got from your mentor over the past year for implementing your laboratory quality improvement plan?_____________**
2. **So overall, how beneficial was the APHL/CDC SEE QA mentorship project to implementing your quality improvement implementation plan?**
3. Not beneficial**, 2.** Somewhat beneficial**, 3.** Very beneficial**, 4.** Critical
4. **Is there anything that you would change to make the mentorship that you received more beneficial for your laboratory?** (in reference to selections above)

***Probe: Can you give an example or explain?*** __________

1. **Were there any other resources or partners that helped you to successfully complete action steps outlined in your plan?**

***Probe:*** ***Who/what resources? How did these partners help you?***

**Long term results of the project & progress towards National Influenza Center requirements and/or designation**

***[Interviewer: Let’s talk just a little more about the impact of the project & progress towards achieving or maintaining lab functions that are required of National Influenza Centers]***

1. **To what extent has mentorship helped you address NIC requirements? [REFER TO NIC spreadsheet] 1.** Extensively**, 2.** Somewhat**, 3.** A little

***Probe: Can you tell me a little more about that & how it made a difference? ________***

***Prove: Was there anything that participating in the project did to help you with areas of concern over the past year?***

1. **Were there any specific skills that you gained from the participation in the APHL/CDC SEE QA mentorship project?**

***Probe: What type of skills?***

***Probe: How did you gain those skills….was it through the mentor or outside of this program?***

1. **Before the mentoring project, how able was your laboratory to identify & address quality issues**?

***So on a scale from 1 to 4, you’d say it was***

**1** Very difficult, **2** somewhat difficult, **3** somewhat easy and **4** very easily
***Probe: Can you give me an example (of things you had identified prior or would have missed prior)?***

1. **Now, after 1 year of mentorship, how able is your laboratory to identify & address quality issues**?

***So on a scale from 1 to 4, you’d say it was***

**1** Very difficult, **2** somewhat difficult, **3** somewhat easy and **4** very easily

***Probe: Can you give me an example (of things you have identified recently or would be looking for)?***

1. **Did any of the improvements over the past year lead to broader quality improvement, beyond influenza diagnostics?**

***Probe: what type of improvements?***

***Probe: what % of activities made broader improvements (<50%, >50%, 100%)***

1. **What was the biggest impact the APHL/CDC SEE QA mentorship project made?______________________________________________**
2. **In terms of addressing laboratory quality improvement, how impactful do you think mentorship through this project was on a scale from 1 to 4, 1 being not impactful and 4 being critical?**

**1**. Not Impactful **2.** Somewhat impactful **3.** Very impactful **4.** Critical

**What lessons have been learned about the technical mentorship model for laboratory quality improvement that could be used to improve the program?**

1. **If this mentorship program were to be re-created, what changes or improvements would you suggest based on your experience? What would you like to stay the same?**

**---------------------------------------------------------------------------------------------------------------------**

**Questions for Mentors:**

**Development of the quality improvement plan**

**[Interviewer: *So I understand that you have been working on completing action steps over the past year. I want to talk a little bit about the development of the quality improvement plan, to learn what worked well and how we might be able to improve the process in the future*]**

1. **How did you and your mentee decide which action steps to focus on over the past year?**

***T*ransition*: In regards to the LQSI training & assessment that you participated in…]***

1. **Did you notice any advantages to your mentee using the LQSI training to develop the quality improvement implementation plan? ________**

***Probe: Can you tell me a little bit more about that?***

***Probe: So everything was clear to you? And everything seemed relevant?***

**What about any disadvantages?**

1. **Were there any other assessments, evaluations, or internal laboratory findings results that helped you and the mentee identify & prioritize action steps for their implementation plan?**

***Probe: Can you tell me a little bit more about that?***

***Transition: If CDC/APHL assessment NOT mentioned, introduce otherwise, transition…***

**3b. Was the CDC/APHL International Influenza Laboratory Capacity Assessment done before or after making your action plan?...how much did you & your mentee rely on those recommendations in prioritizing activities over the past year?**

***Probe: Can you tell me a little bit more about that…what parts were helpful? Why you didn’t/did use it?***

1. **So how likely would it have been for the lab to develop and make progress on a quality improvement plan without the APHL/CDC SEE QA mentorship provided?**

***Probe: In what ways did the project help you prioritize action steps?***

**Connection to and follow-up with mentee and APHL over the course of the year**

***[Interviewer: It would be nice to talk a little bit about how you and your mentor have communicated and followed up over the past year]***

1. **How would you describe the follow-up between you and your mentor over the past year**?

**Too Frequent, Just Right, Not Frequent Enough**

***Probe: Why was that? Who reached out to who first?***

**5a. So about how often did you and your mentor communicate (on average)?**

**Weekly, Every Few Weeks, Monthly, As needed**

1. **About how often did you and your mentee communicate (on average)?**

**Weekly, Every Few Weeks, Monthly, As needed?**

***Probe: who typically initiated communication? For what reason?***

1. **What was the best way to communicate with your mentee over the past year?**

**Email , Skype, Telephone, Other?**

***Probe: Why was that?***

***Probe: What types of challenges did you experience?***

1. **How helpful was the monthly check-in with your mentee to review & make progress on action steps for your laboratory?**

***Probe: what would have made that process better for you?***

***Probe: is there anything you’d change about the process more helpful?***

***Probe: is there anything you’d change to make the process more helpful?***

1. **How useful were the monthly reports you received from your mentee?**

***Probe: why?***

**9a. So on a scale from 1 to 4, 1 being useless and 4 being very useful, you’d say the reports were… 1.** very difficult**, 2.** Somewhat difficult, **3.** fairly easy**, 4.** Very easy

***Probe: why?***

***Probe: Can you think of anything that would make reporting to your mentor easier, clearer, better, or more useful for you?***

1. **Is there anything that you would change about the follow-up processes with your mentee?**

***Probe: How helpful were the monthly reports? The quarterly calls with all mentors? Are there things you would change about either?***

**10a. In regards to year 2, do you think a standard, reporting template improve the utility of information you receive?**

**Implementation guidance & support**

***[Interviewer: Now I’d like to talk about implementation of the plan & how you supported and guided your mentee***

1. **Did you place any documents on EZCollab over the past year?**

***Why?***

***Probe: Can you give an example or explain?*** __________

1. **Were there any documents/resources that you shared outside of EZCollab that helped in the development of your own material?**

***Probe: How often did they share documents outside of the EZCollab system?***

***Probe: Was it as you requested them or did they share things proactively?***

1. **Were there any specific documents that helped in the development of your own material?**

***Probe: what were they? Did you access them on EZCollab or were they sent directly?***

1. **What were some of the challenges you faced in helping your mentee to implement the quality improvement plan?**

***Probe: were the challenges expected?***

***Probe: What helped you to overcome the challenges? ___________***

1. **Can you think of anything about that helped your mentee with expected or unexpected challenges?**

***Probe: Can you give an example or explain?*** __________

1. **Were there any other resources or partners that helped you with project implementation?**

***Probe:*** ***Who/what partners/resources? How did these partners help you?***

1. **So overall, how beneficial was the APHL/CDC SEE QA mentorship project to implementing the quality improvement plan?**
2. Not beneficial**, 2.** Somewhat beneficial**,3.** Very beneficial, **4.** Critical
3. **Is there anything that you would change to make the mentorship that you gave more beneficial?** (in reference to selections above)

***Probe: Can you give an example or explain?*** __________

1. **Were there any other resources or partners that helped you to successfully mentor?**

***Probe:*** ***Who/what resources? How did these partners help you?***

**Long term results of the project & progress towards National Influenza Center requirements and/or designation**

***[Interviewer: Now we’re going to talk just a little bit more about the impact of the project & progress towards achieving or maintaining lab functions that are required of National Influenza Centers]***

1. **Which NIC requirements are you most concerned about improving [or maintaining (Albania)] in the countrie(s) you have worked with on this project?**

**Virus isolation:** …. ***Probe: why is that?***

**Virus Shipment: Sending timely samples to WHO CCs for seasonal vaccine strain selection**… ***Probe: why***

**Influenza virus characterization**… ***Probe: why is that?***

**Other**__________________________________________________

1. **To what extent do you feel the project facilitated progress in NIC functions over the past year?**

**Extensively, Somewhat, A little**

***Probe: Can you tell me a little more about that & how it made a difference? ________***

1. **Before the mentoring project, how would it have been for the lab to identify & address quality issues**?

***So on a scale from 1 to 4, you’d say it was***

**1** Very difficult, **2** somewhat difficult, **3** somewhat easy and **4** very easily

***Probe: Can you give me an example (of things you had identified prior or would have missed prior)?***

1. **Now, after 1 year of mentorship, how would it have been for the lab to identify & address quality issues**?

***So on a scale from 1 to 4, you’d say it was***

**1** Very difficult, **2** somewhat difficult, **3** somewhat easy and **4** very easily

1. ***Probe: Can you give me an example (of things you have identified recently or would be looking for)?*Did any of the improvements over the past year lead to broader quality improvement, beyond influenza diagnostics?**

***Probe: what type of improvements?***

1. **What was the biggest impact that the APHL/CDC SEE QA mentorship project made?______________________________________________**
2. **In terms of addressing laboratory quality improvement, on a scale from 1-4, how impactful do you think mentorship through this project was?**

**1. Not Impactful 2. Somewhat impactful 3. Very impactful. 4. Critical**

**What lessons have been learned about the technical mentorship model for laboratory quality improvement that could be used to improve the program?**

1. **If this mentorship program were to be re-created, what changes or improvements would you suggest based on your experience and lessons learned? What would you like to stay the same?_______________________________________________________________**

***Probe: For mentors? For countries/mentees? For project organizers/partners?***

**---------------------------------------------------------------------------------------------------------------------**

**Questions for Partners: SECID & WHO**

**Partnership between APHL, WHO, CDC, KIT, SECID, and National Influenza programs in the target countries**

1. **Can you tell me about the history of the project?**

***Probe: What has SECID’s main contribution to the project been over the last year?***

1. **What types of partnerships do you feel have been important for supporting your activities toward laboratory quality improvement in the region? Why?**

**[interviewer]*: we also wanted to talk a little about the development of the plan for quality improvement]***

**Development of the quality improvement plan**

1. **So overall, how beneficial do you think the APHL/CDC SEE QA mentorship project was for countries in the region in terms of developing an implementation plan for quality improvement?**

**1*.* Not beneficial, 2. Somewhat beneficial, 3. Major, 4. Critical**

***Probe: Can you tell me a little about that?***

**Follow-up over the course of the year**

***[Interviewer: I’d like to talk a bit about communication and followed up over the past year]***

1. **How would you describe the follow-up process between yourself, APHL, and CDC over the past year?**

**Too frequent, Just right, Not frequent enough**

***Probe: Why was that?***

***Probe: How often was it?***

**Weekly, every few weeks, monthly, as needed**

1. **How helpful was the follow-up over the course of the year for understanding progress in the region & how to assist with implementation of the project?**

***Probe: what would have made that process***

***Probe: is there anything you’d change about the process more helpful?***

***Probe: so you wouldn’t change anything about that process?***

1. **Is there anything that you would change about the follow-up process to make it easier for you to contribute to laboratory quality improvement actions in countries?**

**Implementation guidance & support over the course of 1-year**

***[Interviewer: Now I’d like to talk about implementation guidance & support for the project over the last year***

1. **What were some of the major challenges that laboratories faced in the region over the past year?**
2. **Can you think of anything that this project has facilitated to help with those challenges?**

**Long term results of the project & progress towards National Influenza Center requirements and/or designation**

***[Interviewer: Now we’re going to talk about impact of the project and lessons learned]***

1. **Which NIC functions are you most concerned about improving in the region? Why?**

**Virus isolation**…. ***Probe: why is that?***

**Sending timely samples to WHO CCs for seasonal vaccine strain selection**… ***Probe: why?***

**Influenza virus characterization**… ***Probe: why is that?***

**Other**__________________________________________________

1. **To what extent has the APHL/CDC SEE QA mentorship project helped to make improvements to this area over the past year?**

**Not at all, A little, Somewhat, Extensively**

***Probe: Can you tell me a little more about that & how it made a difference? ________***

1. **What was the biggest impact that the APHL/CDC SEE QA mentorship project made?______________________________________________**
2. **In terms of addressing laboratory quality improvement, on a scale from 1-4, how impactful do you think the APHL/CDC SEE QA mentorship project was?**

**1. Not impactful 2. Somewhat impactful 3. Very impactful 4. Critical**

**Lessons learned about the technical mentorship model for laboratory quality improvement that could be used to improve the program**

1. **If this mentorship program were to be re-created, what changes or improvements would you suggest based on your experience and lessons learned? What would you like to stay the same?______**
